# IL-33/soluble ST2 axis is associated with radiation-induced cardiac injury

**DOI:** 10.1515/biol-2022-0841

**Published:** 2024-03-30

**Authors:** Xiaokeya Yasen, Renaguli Aikebaier, Atiguli Maimaiti, Munire Mushajiang

**Affiliations:** Department of Tumor Internal Medicine, The First People’s Hospital of Kashgar Prefecture, Xinjiang, China; Department of Breast Radiotherapy, Cancer Hospital Affiliated to Xinjiang Medical University, 789 Suzhou East Street, Xinshi District, Urumqi City, Xinjiang 830000, China

**Keywords:** IL-33, sST2, cardiac damage, radiation, breast cancer, TCF7L2

## Abstract

Radiotherapy for treating breast cancer is associated with cardiac damage. This study aimed to investigate the role of the interleukin (IL)-33/soluble receptor ST2 (sST2) axis in radiation-induced cardiac injury. Expressions of IL-33 and sST2 were detected in breast cancer patients following radiotherapy, radiation-induced cardiac damaged mice model, and cardiomyocytes using quantitative real-time PCR (qRT-PCR) and immunohistochemical assay. Cardiac injury was evaluated through an ultrasound imaging system and hematoxylin & eosin staining. The transcriptional factor was assessed using dual-luciferase reporter assay and chromatin immunoprecipitation. The results indicated that IL-33 and sST2 were highly expressed in breast cancer patients, which further elevated post-6 months but reduced after 12 months of radiotherapy. Radiation induces cardiac dysfunction and elevated IL-33 and sST2 levels in a time-dependent manner. However, silencing of IL-33 decreased sST2 expression to alleviate radiation-induced cardiac dysfunction. The IL-33 could be transcriptional activated by TCF7L2 by binding to IL33 promoter sites, which mutation alleviated cardiomyocyte injury caused by radiation. Additionally, radiation treatment resulted in higher levels of TCF7L2, IL-33, and sST2 in cardiomyocytes, and TCF7L2 knockdown reduced IL-33 and sST2 expression. In conclusion, TCF7L2 transcriptional-activated IL-33 mediated sST2 to regulate radiation-induced cardiac damage, providing novel insights into radiotherapy-induced cardiac damage.

## Graphical abstract

**Figure:**
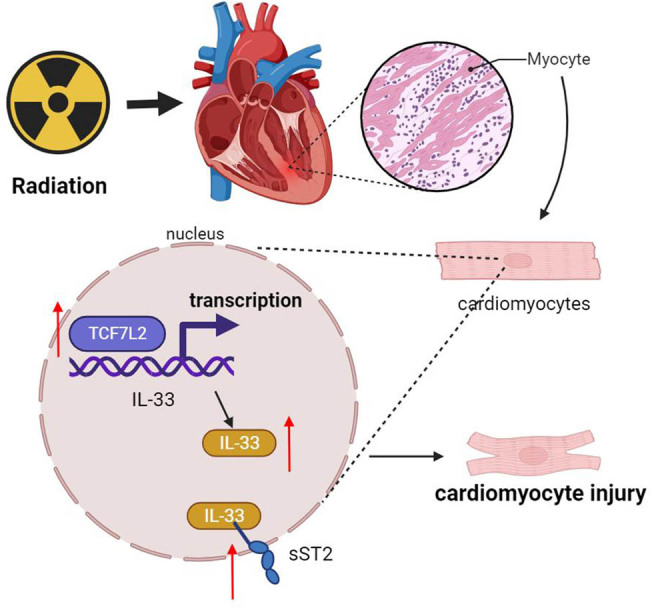

## 1 Introduction

Breast cancer remains the leading cause of cancer-associated death in women worldwide [1]. The treatment strategy for breast cancer has enormously improved in recent years, and radiotherapy has proven effective in controlling the disease and improving survival rates [2]. Regional lymph node irradiation provides better coverage of the target area and reduces long-term toxicity in patients [2]. Nonetheless, the heart is often exposed during breast cancer radiotherapy, thereby elevating the risk of radiation-associated cardiac damage [3]. Generally, the heart receives a dose of 1–5 Gy; however, regional lymph node radiation usually results in a higher dose to the heart, inducing non-breast cancer-related death [4,5]. Although not absolute, the heart-exposed dose is associated with the development of cardiovascular disease [4,6] Therefore, it is imperative to elucidate the underlying mechanisms of radiation-induced heart damage and to provide novel preventive strategies.

Interleukin (IL)-33, a tissue-derived nuclear cytokine, is a member of the IL-1 family expressed in endothelial, epithelial, and fibroblast cells [7], playing a vital role in cell homeostasis, immune response, and tissue regeneration [8,9]. IL-33 is upregulated in barrier sites and serves its functions by mediating the suppression of tumorigenicity 2 (ST2), encoded by IL-1 receptor like-1 (IL-1RL1) [10]. The ST2 belongs to the IL-1 receptor family and exists in two isoforms, namely transmembrane (ST2L) and soluble (sST2) [11]. It has been reported that the IL-33/ST2 axis was involved in several pathologic processes, including organ fibrosis [12], autoimmune disease [13], cancers [14], and nerve injury [15]. IL-33 and sST2 are both associated with the development and metastasis of breast cancer [16,17]. Additionally, IL-33 has also been found to have cardioprotective effects, which are inhibited by sST2, a decoy receptor [18]. However, whether IL-33 is involved in cardiac injury after radiotherapy by regulating the sST2 receptor has not been elaborated.

This study investigated the involvement of the IL-33/sST2 axis in radiation-induced cardiac injury to provide potential novel targets for treating cardiac damage post-radiotherapy in breast cancer patients.

## 2 Materials and methods

### 2.1 Subjects

A total of 300 subjects were enrolled in this study, among which 50 were healthy individuals, 50 patients with breast cancer before radiotherapy, 50 patients who received radiotherapy once, 50 patients post 1 month of radiotherapy, 50 patients post 6 months of radiotherapy, and 50 patients post 12 months of radiotherapy. All patients had tumors in their left breast and had not received any other treatments. Whole blood samples were collected from all subjects, allowed to stand for 30 min, and then centrifuged at 3,000 rpm for 10 min to collect the serum, which was then stored at −80°C until further use. The clinical information of patients with breast cancer is shown in Table 1.

**Table 1:** Clinical details of patients with breast cancer

Parameters	Number
Age (median with range)	51 (23–84)
**pT stage (tumor size)**	
pTis	15
pT1 (≤2 cm)	123
pT2 (2 < *T* ≤ 5)	104
pT3 (>5)	8
**Lymph node metastasis (N stage)**	
pN0 (0)	125
pN1 (1–3)	60
pN2 (4–9)	52
pN3 (≥10)	13
**Estrogen receptor status**	
Negative	41
Positive	209
**Progesterone receptor status**	
Negative	48
Positive	202
HER2 status	
Negative	172
Positive	48
Unknown	30
**Clinical TNM stage**	
0	11
I	61
II	95
III	83


**Informed consent:** Informed consent has been obtained from all individuals included in this study.
**Ethical approval:** The research related to human use has been complied with all the relevant national regulations, institutional policies and in accordance with the tenets of the Helsinki Declaration, and has been approved by the Ethics Committee of Cancer Hospital Affiliated to Xinjiang Medical University.

### 2.2 Animal model establishment

C57BL/6 mice (4–6 weeks old, 20 ± 2 g, female) were purchased from Charles River (Beijing, China). After 1 week of acclimatization, the mice were divided into five groups (*n* = 6/group): control, RT, RT + 1W, RT + 2W, and RT + 4W. The mice in the control group were anesthetized by intraperitoneal injection of 2% isoflurane with sham radiation and euthanized 4 weeks later, while other group animals were used to establish the radiation-induced heart damage (RIHD) model. Briefly, the mice were anesthetized using the same method as the control, immobilizing their limbs in the supine position, and their heart was irradiated with a 6 MV energy X-ray beam with 14 Gy/1 Fx dose using a medical linear accelerator (Varian Trilogy, FL, USA) as previously described [19]. The mice were euthanized with radiation for once, and 1, 2, and 4 weeks of radiation.

To explore the role of IL-33 *in vivo*, short hairpin RNA (sh)-IL-33 and its negative control (sh-NC) were synthesized by GenePharma (Shanghai, China). The mice were divided into four groups (*n* = 6/group): control, RT + 2W, RT + 2W + sh-NC, and RT + 2W + sh-IL-33. sh-IL-33 and sh-NC were packaged in lentivirus. The mice were anesthetized by 2% isoflurane to expose the hearts. Lentivirus (10 μL, 10^9^ PFU/mL) were administrated through vein injection for 3 consecutive days. Then, the mouse hearts were irradiated with X-ray for 2 weeks.


**Ethics statement:** The research related to animal use has been complied with all the relevant national regulations and institutional policies for the care and use of animals and has been approved by the Ethics Committee of Cancer Hospital Affiliated to Xinjiang Medical University.

### 2.3 Cardiac function analysis

Cardiac function was measured before euthanasia. The left ventricular ejection fraction, left ventricular end-diastolic dimension (LVEDD), and systolic left ventricular diameter (SLVD) were assessed by transthoracic ultrasound using a Vevo2100 high-resolution ultrasound imaging system (VisualSonics, USA).

### 2.4 Hematoxylin & eosin (H&E) staining assay

Following euthanasia, the heart from all animals was surgically excised after perfusing 20 mL phosphate buffer saline (PBS) through systemic circulation, followed by fixation in 4% polyformaldehyde and sliced into 4 μm paraffin sections using a microtome. The sections were subjected to H&E staining for assessing tissue pathology, where after dewaxing and dehydration, the sections were stained with hematoxylin (Aladdin, Shanghai, China) for 5 min, washed, and counterstained with 0.5% eosin (Aladdin, Shanghai, China) for 3 min.

### 2.5 Immunohistochemical (IHC) assay

The heart tissue sections were dewaxed and rehydrated, and endogenous peroxidase activity was eliminated using 3% hydrogen peroxide, followed by blocking them with normal goat serum in PBS. The sections were then incubated overnight with primary antibodies against IL-33 and sST2 at 4°C, followed by incubation with a secondary antibody for 30 min at 37°C. Diaminobenzidine solution (Aladdin, Shanghai, China) was employed for 10 min for displaying sections color. After washing in running water, the sections were dehydrated, permeated, sealed, and viewed under a light microscope.

### 2.6 Cell culture and radiation

Rat cardiomyocytes (H9C2) were purchased from the ATCC and maintained in Dulbecco’s modified eagle’s medium (Hyclone, South Logan, UT, USA) containing 10% fetal bovine serum (Hyclone) and 1% penicillin/streptomycin at 37°C with 95% air and 5% CO_2_. The cells received the 6 MV X-ray beam energy with 0, 2, 4, and 8 Gy using a medical linear accelerator for 48 h. Additionally, the cells received 8 Gy X-rays for 0, 12, 24, and 48 h. Radiation parameters were set as previously described [19].

### 2.7 Quantitative real-time PCR (qRT-PCR)

Total RNA was isolated from the serum of patients with breast cancer, mouse heart, and H9C2 cells using TRIzol reagent (Invitrogen, ThermoFisher Scientific, MA, USA). The cDNA was synthesized using the TaqMan™ Reverse Transcription Reagents (Invitrogen). Then, qPCR was carried out using the ABsolute qPCR low ROX Mix (ThermoFisher Scientific) on the ABI PRISM 7500 system according to the manufacturer’s protocol. The reaction mixture contains Absolute blue SYBP Green ROX (12.5 μL), forward primer (1.75 μL), reverse primer (1.75 μL), cDNA (2 μL), and nuclease-free water (7 μL). The reaction conditions were 95°C for 15 min, 40 cycles of 95°C for 15 s, 56°C for 30 s, and 72°C for 30 s. GAPDH served as the control. The mRNA expression was calculated by the 2^−ΔΔCt^ method. The primer sequences are shown in Table 2.

**Table 2:** Specific primer sequences used in qRT-PCR

Name	Species	Sequences (forward 5ʹ–3ʹ)	Sequences (reverse 5ʹ–3ʹ)
IL-33	Human	GGTGACGGTGTTGATGGT	TGGTCTGGCAGTGGTTTT
sST2	Human	CAGGAAAGAAATCGTGTGT	GCCAAGAACTGAGTGCCT
IL-33R	Human	CTCTACAACTGGACAGCACCTC	TGCGTCCTCAGTCATCACAT
IL-1RAcP	Human	AGTGATGCCTCAGAACGC	TGGGCTGTGCTGTAGTTG
GADPH	Human	GACCTGACCTGCCGTCTA	AGGAGTGGGTGTCGCTGT
IL-33	Mouse	ATTTCCCCGGCAAAGTTCAG	AACGGAGTCTCATGCAGTAGA
sST2	Mouse	TGACACCTTACAAAACCCGGA	AGGTCTCTCCCATAAATGCACA
GAPDH	Mouse	AGGTCGGTGTGAACGGATTTG	TGTAGACCATGTAGTTGAGGTCA
TCF7L2	Rat	AAGTGCGTTCGCTACATACA	CAGAGGATCAGGCTTCAGG
IL-33	Rat	GACAGCACATCAGGCAGAG	TGAGGCCAGAACGGAGC
sST2	Rat	AGCGCCTGTTCAGTGGTT	TGGTTCCGTTCTCCGTGT
GAPDH	Rat	AACTCCCTCAAGATTGTCAGC	GAGCCCTTCCACGATGC

### 2.8 Western blotting

Total proteins were extracted from mouse heart tissues and H9C2 cells using RIPA lysis buffer. Proteins from the serum of participants were extracted using the serum protein extraction kit (Solarbio, Beijing, China). After concentration was measured using a BCA protein assay kit (Solarbio), the protein samples were run on 15% SDS-PAGE and transferred to polyvinylidene fluoride membranes. After blocking, the membranes were incubated with primary antibodies targeting IL-33 (ab118503; Abcam, Cambridge, UK), sST2 (ab25877; Abcam), TCF7L2 (sc-271287, Santa Cruz Biotechnology, Santa Cruz, CA, USA), GAPDH (ab9485; Abcam) at 4°C overnight, followed by incubation with anti-rabbit (ab6721; Abcam) or anti-mouse (ab205719; Abcam) conjugated to horse radish peroxidase at 25°C for 1 h. The band signals were visualized using an ECL substrate (Solarbio).

### 2.9 Bioinformatic analysis

The DNA motif of TCF7L2 and the binding sites between TCF72L and IL33 promoter were predicted by the JASPAR online tool (http://jaspar.genereg.net/).

### 2.10 Chromatin immunoprecipitation (CHIP)

A CHIP assay kit was purchased from Beyotime (Shanghai, China). H9C2 cells were cross-linked with 1% methanol for 10 min at 37°C, followed by washing with 1 mM PMSP in PBS and lysed using the SDS lysis buffer containing 1 mM phenylmethanesulfonyl fluoride for 10 min. The DNA was cleaved with ultrasonic treatment, followed by centrifuging and diluting samples using a ChIP dilution buffer. It was proceeded by incubating samples with Protein A + G Agarose/Salmon Sperm DNA for 30 min at 4°C. After centrifugation, the supernatant was incubated with anti-TCF7L2 and anti-IgG antibodies overnight at 4°C. Afterward, washing with low and high salt, and LiCl immune complex buffers in sequence, the enrichment of IL-33 was detected using qRT-PCR.

### 2.11 Luciferase reporter analysis

IL33 promoter sequences and mutant sequences were amplified and inserted into the pmiR-GLO vector (Promega, Madison, WI, USA). The H9C2 cells were seeded into 24-well plates and co-transfected the wild-type or mutant sequences recombinant plasmids (lipofectamine 2000) along with TCF7L2 overexpressing and empty vectors. Following 48 h, the luciferase activity was detected using the dual-luciferase reporter assay system (Promega).

### 2.12 Cell transfection

H9C2 cells were seeded in six-well plates and cultured until 80% cell confluence. Sh-TCF7L2 and sh-NC were purchased from Genepharma. These vectors were transfected into H9C2 cells using lipofectamine 3000 (Invitrogen). After 48 h, cells were harvested and TCF7L2 expression was measured using qRT-PCR.

### 2.13 Determination of lactic dehydrogenase (LDH) activity

LDH activity in H9C2 cells were measured using a LDH activity assay kit (Elabscience, Wuhan, China). Briefly, H9C2 cells were homogenated in PBS. After centrifuging at 10,000*g* for 10 min, the supernatant was collected. The protein concentration was detected using a BCA kit (Elabscience). The supernatant (20 μL) was incubated with 25 μL substrate buffer and 5 μL coenzyme I at 37°C for 15 min. Next, 25 μL chromogenic agent was added to incubate with the mixture at 37°C for 15 min. After adding alkali reagent, the absorbance was measured using a microplate reader.

### 2.14 Statistical analysis

All data were analyzed with one-way ANOVA among multiple groups using the GraphPad Prism 8 statistics software. *P* < 0.05 was considered a significant difference.

## 3 Results

### 3.1 IL-33 and sST2 are dysregulated after radiotherapy for breast cancer

**Figure 1:**
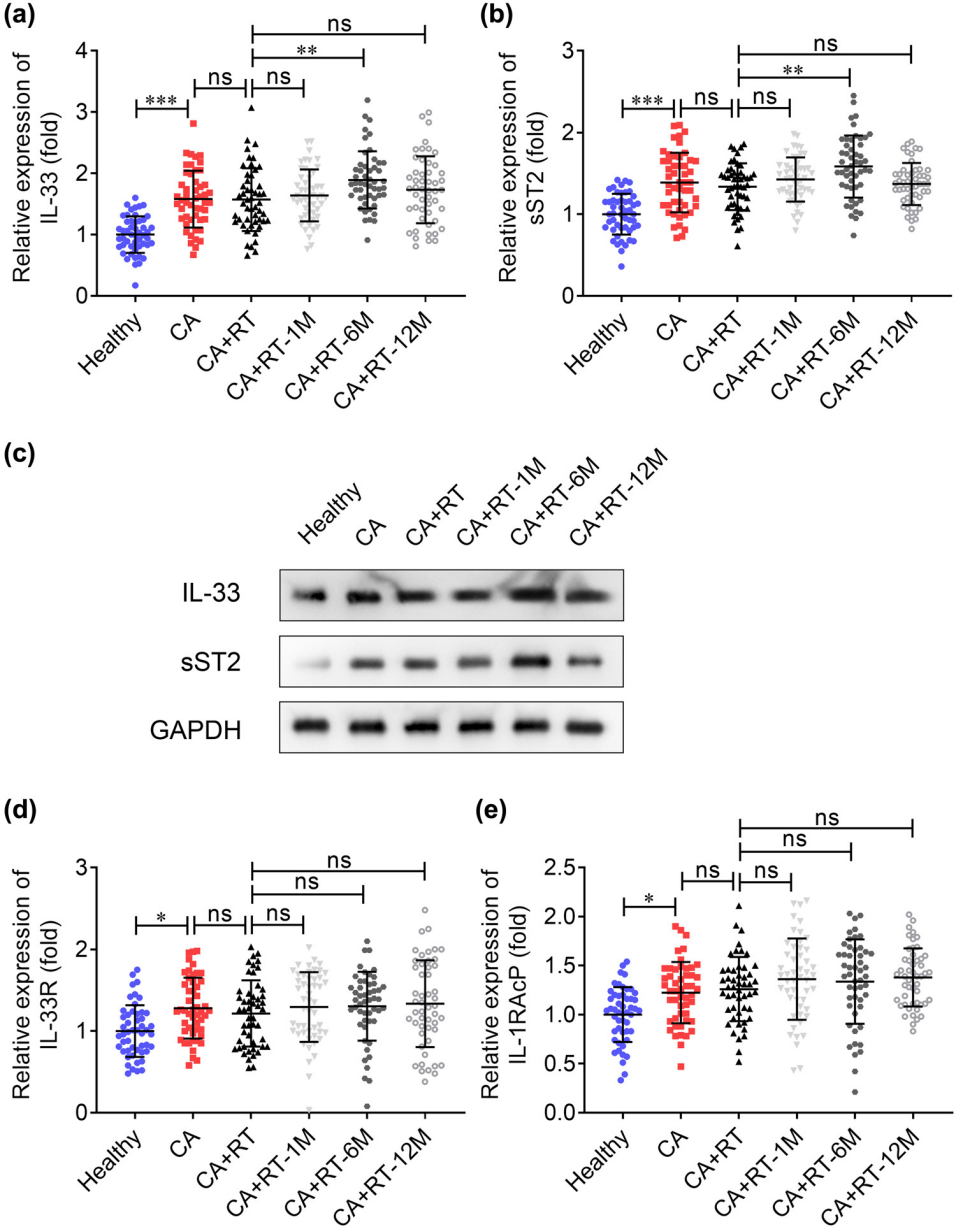
IL-33 and sST2 are dysregulated after radiotherapy for breast cancer. (a) IL-33 and (b) sST2 levels were detected in healthy subjects (*n* = 50), patients with breast cancer before radiotherapy (*n* = 50; CA), radiotherapy for once (*n* = 50; CA + RT), 1 month after radiotherapy (*n* = 50; CA + RT-1M), 6 months after radiotherapy (*n* = 50; CA + RT-6M), and 12 months after radiotherapy (*n* = 50; CA + RT-12M) using qRT-PCR. (c) IL-33 and sST2 protein levels in the serum of each group were measured using western blotting. (d) IL-33R and (e) IL-1RAcP mRNA expression in each group was examined using qPCR. ***P < 0.001; **P < 0.01; ns: no significance.

We enrolled 250 patients with breast cancer and 50 healthy controls in this study. The clinical details of patients are shown in Table 1. The median age was 51 (range from 23 to 84). Most patients had tumors smaller than 5 cm in size. Half of the patients had no lymph node metastasis. Estrogen receptor positive, progesterone receptor positive, and HER2 negative patients accounted for a large proportion of all patients, and TNM stages were mainly concentrated in stage I–III. To explore the function of IL-33 and its receptors, we first examined the levels of IL-33, sST2, IL-33R, and IL-1RAcP in the serum of all subjects. As shown in Figure 1a and b, the levels of IL-33 and sST2 were elevated in patients with breast cancer, compared with the healthy control. Besides, the IL-33 and sST2 levels remained unaltered in patients after receiving radiotherapy once, compared with those without treatment, but were upregulated after 6 months of radiotherapy, and there was no significant difference at 1 or 12 months interval compared to those who received radiotherapy once. The protein levels of IL-33 and sST2 were consistent with their mRNA expression (Figure 1c). Moreover, the IL-33R and IL-1RAcP expressions were elevated in patients with breast cancer compared to healthy control; however, they remained unaffected by radiotherapy (Figure 1d and e).

### 3.2 Radiation induces cardiac dysfunction and the abnormal expression of IL-33/sST2 in RIHD mice

**Figure 2:**
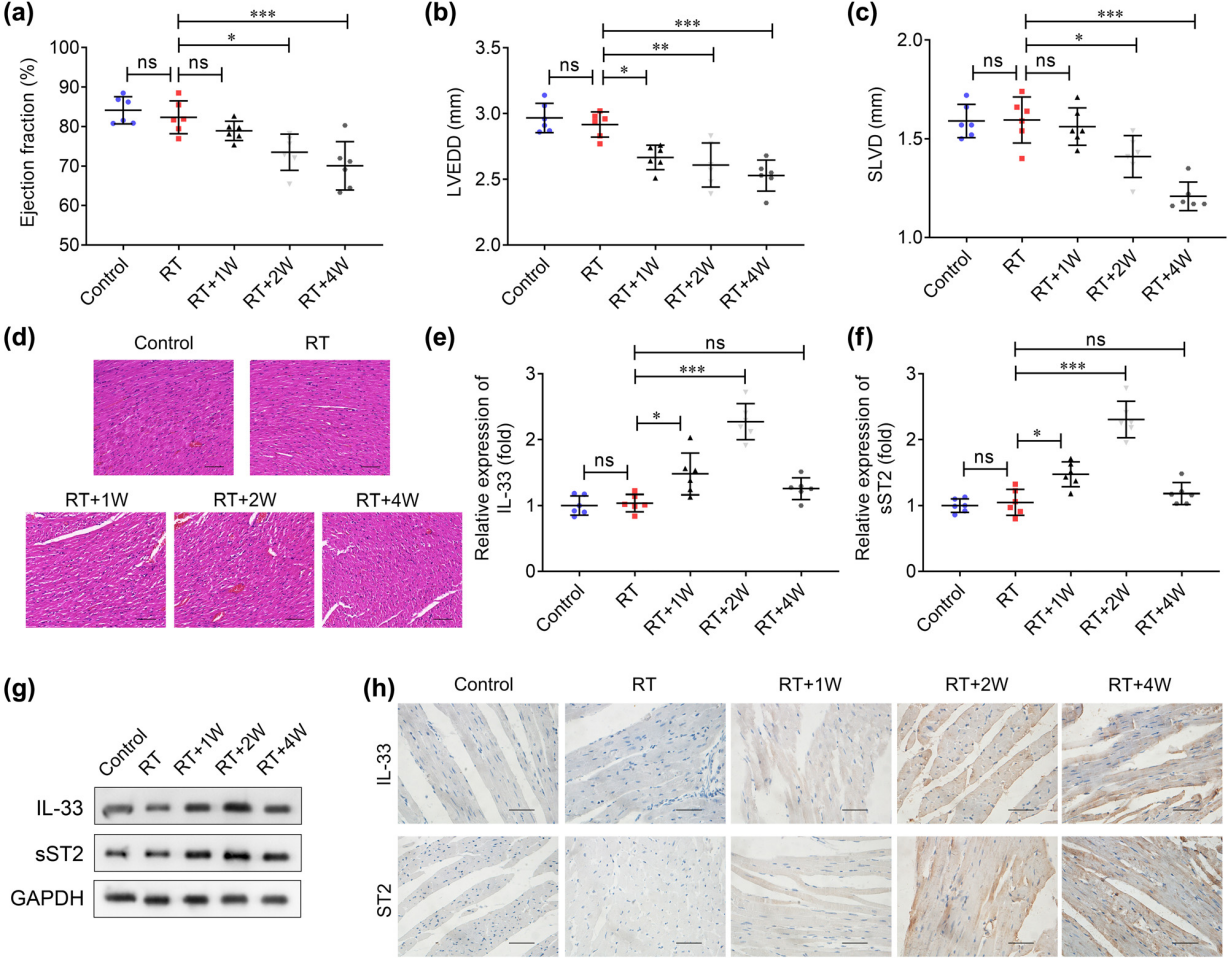
Radiation induces cardiac dysfunction and the abnormal expression of IL-33/sST2 in RIHD mice. The cardiac function including (a) the left ventricular ejection fraction, (b) LVEDD, and (c) SLVD was analyzed using the ultrasound imaging system. (d) Myocardial damage was visualized using the H&E staining assay. Scale bar: 50 μm. (e) IL-33 and (f) sST2 mRNA expression in the hearts of mice. (g) IL-33 and sST2 levels were examined using western blotting in heart tissues of mice. (h) IHC assay showed the protein levels of IL-33 and sST2 in the hearts of mice. Scale bar: 50 μm. ***P < 0.001; **P < 0.01; *P < 0.05; ns: no significance.

The radiotherapy effects on cardiac dysfunction were subsequently assessed in the RIHD animal model. The results showed that the ejection fraction, LVEDD, and SLVD remained unaffected when radiations were received once; however, LVEDD was found reduced when radiotherapy was received for 1 week. The ejection fraction, LVEDD, and SLVD decreased significantly after 2 and 4 weeks of radiation, particularly after 4 weeks (Figure 2a–c). In addition, radiation for 2 and 4 weeks resulted in the irregular arrangement of myocardial cells, local inflammatory infiltration, and myocardial fiber rupturing, indicating myocardial injury (Figure 2d). The mRNA and protein levels of IL-33 and sST2 in heart tissues had no significant difference between the control and RT group. However, compared to mice which received radiation for once, radiation treatment for 1 and 2 weeks upregulated the IL-33 and sST2 levels, while remained unaffected at 4 weeks of radio treatment (Figure 2e–h).

### 3.3 Knockdown of IL-33 reverses the cardiac dysfunction caused by radiation

**Figure 3:**
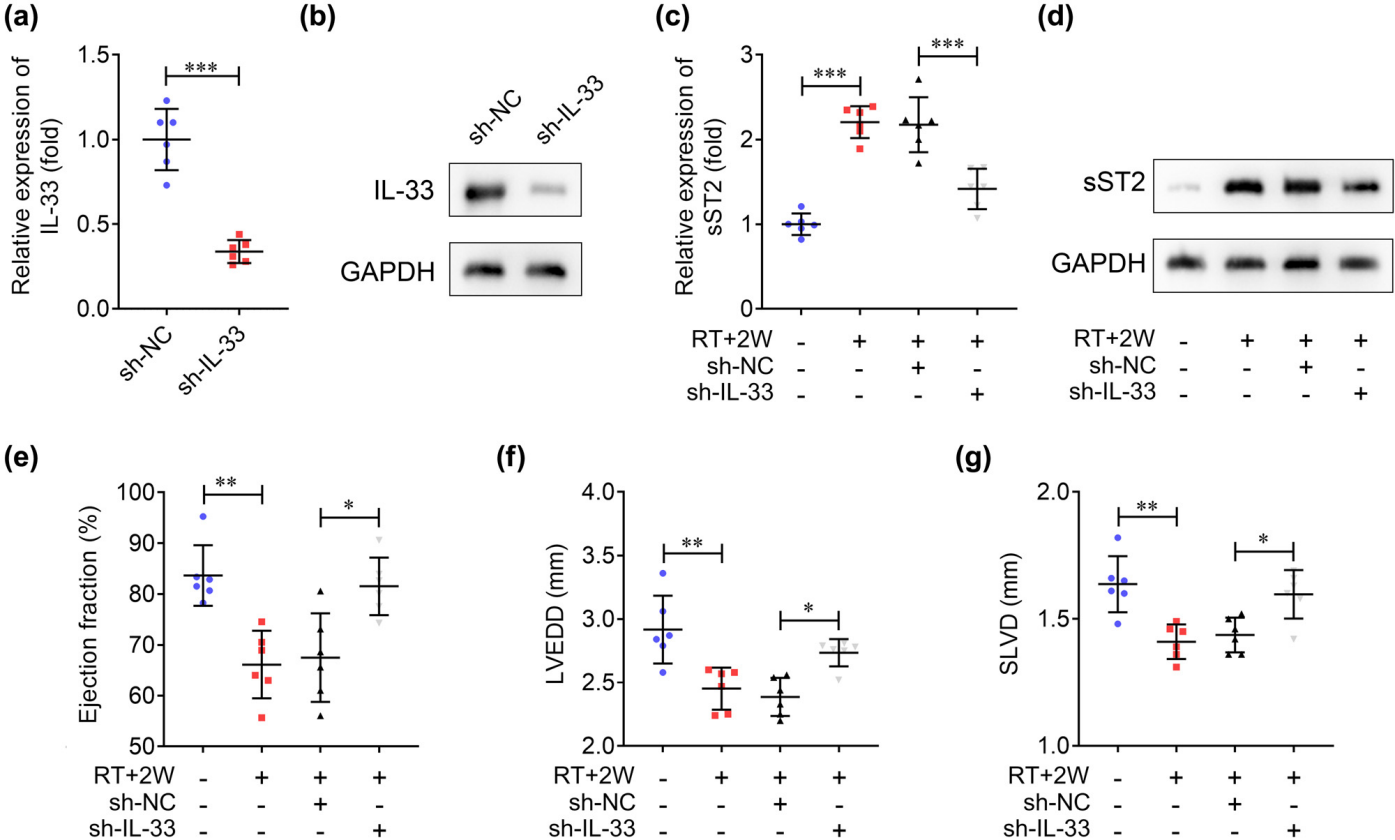
Knockdown of IL-33 reverses the cardiac dysfunction caused by radiation. (a) IL-33 expression in the hearts was measured using qRT-PCR. (b) IL-33 expression in the hearts was measured using western blotting. (c) and (d) sST2 mRNA and protein expression in the hearts of mice after RT stimulation and IL-33 knockdown. (e) The left ventricular ejection fraction, (f) LVEDD, and (g) SLVD were measured to evaluate cardiac function using the ultrasound imaging system. ***P < 0.001; **P < 0.01; *P < 0.05.

To explore the role of IL-33 in cardiac function, we knocked down IL-33 in mice. The expression of IL-33 was reduced after sh-IL-33 injection (Figure 3a and b). Then, we measured sST2 expression in heart tissues. The results showed that RT for 2 weeks increased sST2 expression, while knockdown of IL-33 abrogated this increase (Figure 3c and d). Cardiac function, including ejection fraction, LVEDD, and SLVD, were assessed using transthoracic ultrasound. The results indicated that RT elevated ejection fraction and reduced LVEDD and SLVD, suggesting that RT induced cardiac dysfunction, whereas IL-33 knockdown reversed the effects caused by RT (Figure 3e–g). Talem together, silencing of IL-33 attenuates cardiac injury in RT-induced mice by downregulating sST2 expression.

### 3.4 TCF7L2 promotes IL-33 transcriptional activation

**Figure 4:**
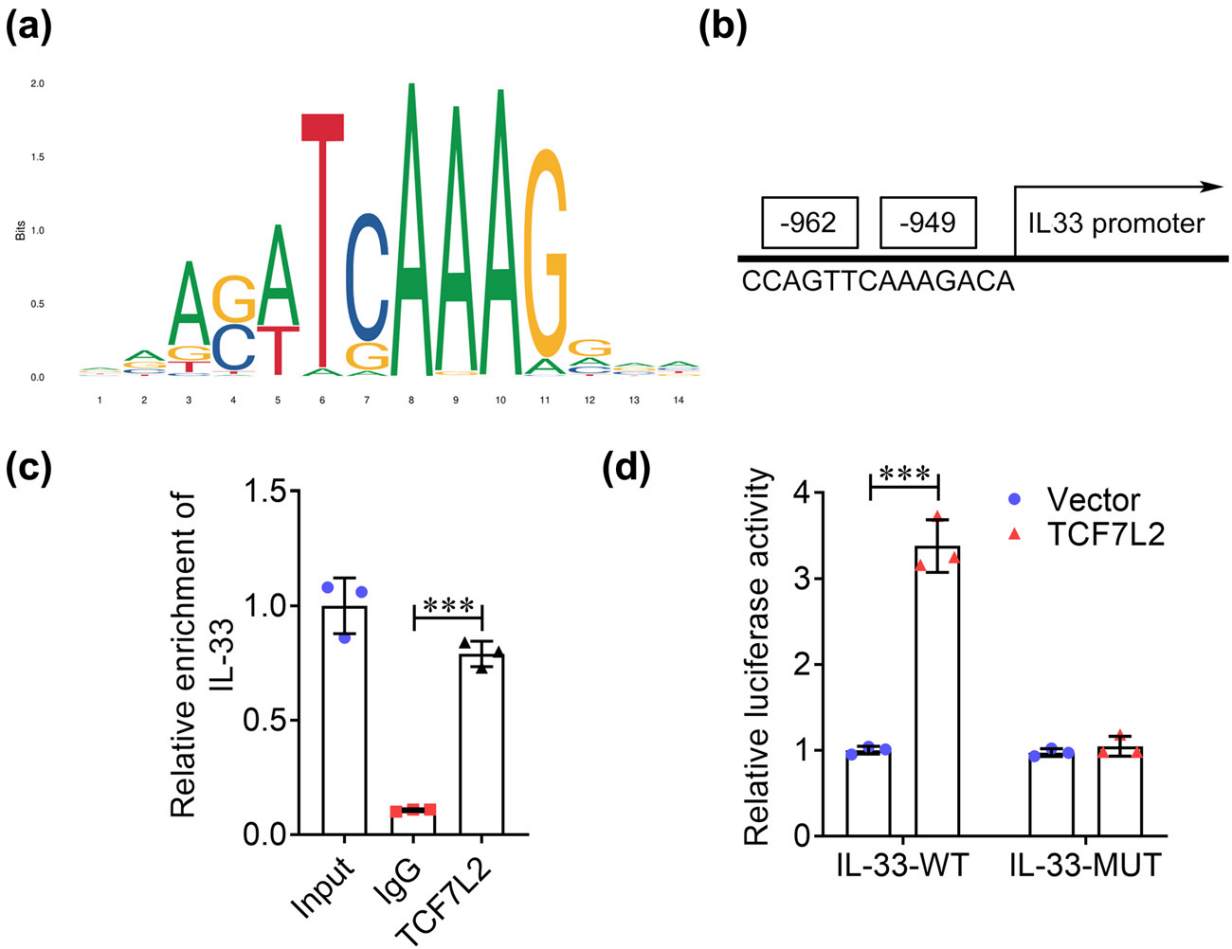
TCF7L2 promotes IL-33 transcriptional activation. (a) The DNA motif of TCF7L2 was predicted using the JASPAR database. (b) The binding sites between TCF7L2 and IL33 promoter were predicted. (c) The affinity of TCF7L2 in IL33 promoter was verified using the CHIP. (d) The binding relationship between TCF7L2 and IL-33 was evaluated using dual-luciferase reporter analysis. ***P < 0.001.

To investigate the factors mediating IL-33 functions, we assessed the upstream transcription factors of IL-33. The TCF7L2 DNA binding motif is shown in Figure 4a, predicting that TCF7L2 could bind to the IL33 promoter (Figure 4b). Overexpression of TCF7L2 increased the enrichment of IL-33, compared with IgG, suggesting that TCF7L2 has an affinity for IL33 promoter (Figure 4c). Besides, the luciferase activity of wild-type IL33 promoter was increased by TCF7L2 overexpression compared to empty vector, whereas TCF7L2 did not affect the luciferase activity of mutant IL33 promoter (Figure 4d).

### 3.5 TCF7L2, IL-33, and sST2 are dysregulated in radiation-induced cardiomyocytes

**Figure 5:**
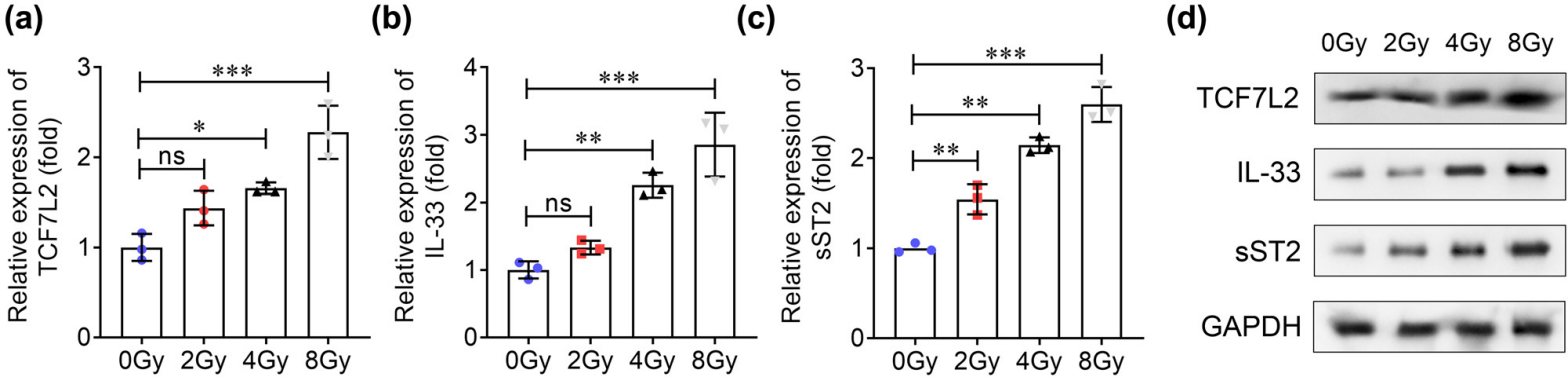
TCF7L2, Il-33, and sST2 are dysregulated in different doses of radiation-treated cardiomyocytes. After H9C2 cells were treated with 0, 2, 4, and 8 Gy X-rays for 48 h, (a) TCF7L2, (b) IL-33, and (c) sST2 levels were detected using qRT-PCR. (d) TCF7L2, IL-33, and sST2 levels in each group were examined using western blotting. ***P < 0.001; **P < 0.01; *P < 0.05; ns: no significance.

**Figure 6:**
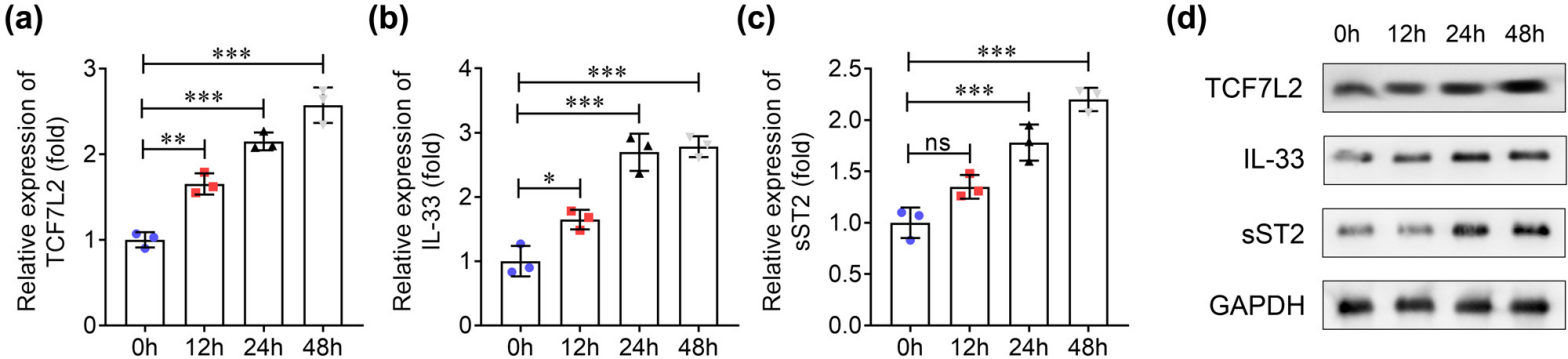
TCF7L2, Il-33, and sST2 are dysregulated in radiation-treated cardiomyocytes at different times. After H9C2 cells were treated with 8 Gy X-ray for 0, 12, 24, and 48 h, (a) TCF7L2, (b) IL-33, and (c) sST2 expression was detected using qRT-PCR. (d) Western blotting was performed to measure TCF7L2, IL-33, and sST2 levels in each group ***P < 0.001; **P < 0.01; *P < 0.05; ns: no significance.

To explore the expression of TCF7L2, IL-33, and sST2 in cardiomyocytes, H9C2 cells were treated with 0, 2, 4, and 8 Gy X-rays for 48 h. Results showed that compared to 0 Gy group, 2 Gy radiation increased sST2 instead of TCF7L2 and IL-33 expressions, whereas 4 and 8 Gy markedly elevated TCF7L2, IL-33, and sST2 expression with an increasing radiation dose (Figure 5a–d). In addition, H9C2 cells were treated with 8 Gy X-ray for 0, 12, 24, and 48 h, and results showed that TCF7L2, IL-33, and sST2 expressions were increased in a time-dependent manner (Figure 6a–d).

### 3.6 Knockdown of TCF7L2 decreases IL-33 and sST2 expression in radiation-induced cardiomyocytes

**Figure 7:**
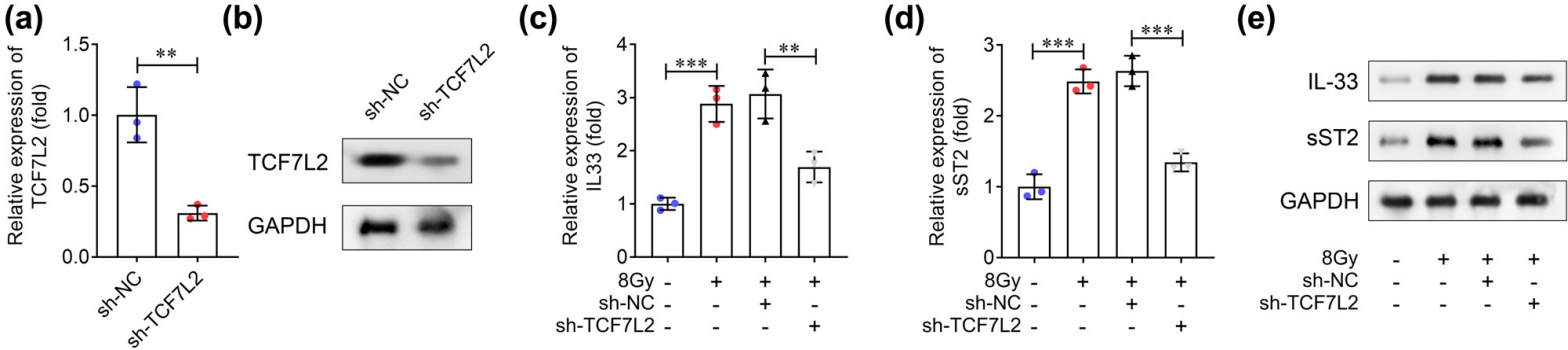
Knockdown of TCF7L2 decreases IL-33 and sST2 expression in radiation-induced cardiomyocytes. (a) and (b) TCF7L2 expression was measured in H9C2 cells after sh-TCF7L2 and sh-NC transfection. (c) IL-33 and (d) sST2 levels were measured using qRT-PCR after cell transfection and 8 Gy X-ray treatment for 48 h. (e) Western blotting was conducted to assess IL-33 and sST2 protein levels. ***P < 0.001; **P < 0.01.

To investigate the effect of TCF7L2 on IL-33 and sST2 expression, TCF7L2 expression was knocked down by transfecting with sh-TCF7L2. The results of qRT-PCR showed that TCF7L2 expression was downregulated after sh-TCF7L2 transfection, compared with sh-NC (Figure 7a and b). Later, IL-33 and sST2 expression was measured using qRT-PCR. The results indicated that 8 Gy X-ray elevated IL-33 and sST2 expression, whereas TCF7L2 knockdown reversed this elevation (Figure 7c–e).

### 3.7 Mutation of IL33 promoter attenuates cell damage

**Figure 8:**
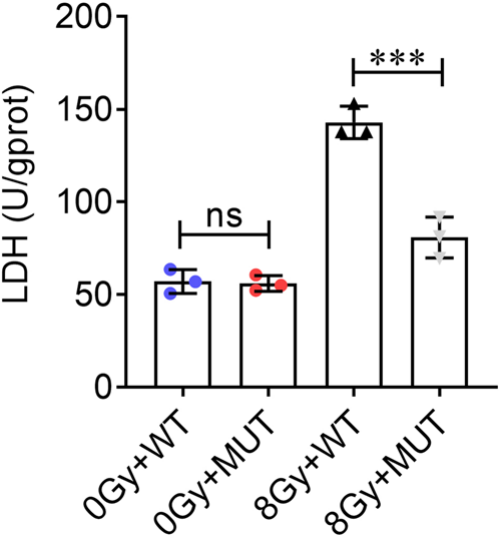
Mutation of IL33 promoter attenuates cell damage. H9C2 cells with wild-type and mutant IL33 promoter were treated with 0 or 8 Gy X-ray, and cell injury was assessed by detecting LDH activity. ***P < 0.001; ns, no significant.

Reporter vectors with wild-type and mutant IL33 promoter sequences were transfected into H9C2 cells, which were treated with 8 Gy X-ray for 48 h. LDH activity was measured to assess cell injury. The results showed that 8 Gy X-ray increased LDH activity. Mutation of IL33 promoter reduced LDH activity after 8 Gy X-ray treatment, but failed to affect LDH activity after 0 Gy X-ray treatment (Figure 8).

## 4 Discussion

Radiotherapy is a commonly used treatment strategy for breast cancer. However, cardiac damage induced by radiation exposure remains a serious concern. Cardiac cells under stress express ST2, whose ligand is reported to be IL-33. The interaction between IL-33 and ST2L has been reported to have a cardioprotective effect; however, when sST2 binds to IL-33 by decoy function, it blocks the interaction between IL-33 and ST2L, resulting in the diminishing of the cardioprotective role [20,21]. They have also been involved in the progression of various cardiovascular diseases, like myocardial fibrosis, hypertrophy, and remodeling [22]. Florens et al. [23] have reported that IL-33 antibody protects heart after acute kidney injury, and overexpression of IL-33 induces cardiac hypertrophy and cardiomyopathy. Asensio-Lopez et al. [24] have found that metformin improves cardiac remodeling after myocardial infarction by increasing IL-33 expression and reducing sST2 levels. These studies indicated the important role of IL-33 and sST2 in heart injury and repair. Meanwhile, IL-33 and sST2 are important tumor development drivers and have been found to promote tumor metastasis in breast cancer [16,25]. Moreover, sST2 has been reported to be used as a biomarker for predicting the cardiac function of breast cancer patients receiving chemotherapeutics [26]. However, whether IL-33 and sST2 regulate radiotherapy-induced cardiac function remains unclear. In this study, the levels of IL-33 and sST2 in patients with breast cancer were elevated compared to healthy subjects, consistent with the previous studies [27,28], suggesting that they play a crucial role in breast cancer development. In addition, radiotherapy for 6 months was found to upregulate IL-33 and sST2 levels, suggesting that radiotherapy for 6 months in breast cancer patients may cause myocardial damage. Moreover, we found an interesting phenomenon that IL-33 and sST2 expressions were decreased after 12 months of radiotherapy compared to 6 months, but not significantly different from 1 month or radiotherapy once. We hypothesized that after 6 months of radiotherapy, IL-33 and sST2 were highly expressed, and they blocked the cardioprotective function of IL-33 through the combination of decoy function, resulting in cardiac injury. However, sustained heart damage may block their binding and promote the heart-protecting effects of IL-33. Therefore, IL-33 and sST2 levels were decreased 12 months after radiotherapy, attenuating heart damage.

To explore the association between cardiac injury and the IL-33/sST2 axis, the RIHD mouse model was established, which is related to the extended follow-up time and partly abolishes the survival benefit of radiotherapy. The incidence of RIHD remains unknown due to the lack of long-term follow-up studies [29]. Our results indicated that cardiac function injury in RIHD mice was gradually severed with increased RIHD time (during 4 weeks). One and 2 weeks of radiation of mice significantly increased the IL-33 and sST2 levels; however, no significant difference was observed at their levels at once and 4 weeks of radiation, suggesting that the IL-33/sST2 axis might be associated with cardiac damage severity as a function of radiation duration. Moreover, we found that knockdown of IL-33 reduced sST2 expression to attenuate cardiac function, suggesting that radiation promotes heart damage by increasing the IL-33/sST2 axis.

TCF7L2 is a transcription factor and is a component of the TCF7L2/β-catenin complex transcriptionally activating the downstream factors in the Wnt pathway [30], and also regulates multiple cellular processes, including cell growth, differentiation, death, and metabolism [31]. The *TCF7L2* gene was reported to be linked with the risk and development of breast cancer and heart diseases [32,33]. Wu et al. [34] have reported that TCF7L2 mediated by miR-509-3p promotes breast cancer cell proliferation and angiogenesis, and inhibits apoptosis. In addition, Li et al. [35] have found that TCF7L2 contributes to anti-atherosclerosis. In this study, we speculated that IL-33 may be regulated by TCF7L2. Our results revealed TCF7L2 transcription-activated IL-33 and a consistent expression trend of TCF7L2, IL-33, and sST2 in irradiated cardiomyocytes. Silencing of TCF7L2 causes sensitivity of cancer cells to X-ray [36]. Thus, we speculated that IL-33 was transcription-activated by upstream TCF7L2 and mediated downstream sST2 to affect radiation-induced heart injury. However, whether TCF7L2 is involved in radiation-induced cardiac dysfunction *in vivo* has not been studied, which will be investigated in our future work.

In conclusion, the results show that the TCF7L2/IL-33/sST2 axis promotes radiation-induced cardiac damage. These findings are envisaged to provide novel information regarding radiotherapy-induced cardiac injury in patients with breast cancer.
